# Multiparameter characterization of subnanometre Cr/Sc multilayers based on complementary measurements

**DOI:** 10.1107/S1600576716015776

**Published:** 2016-11-24

**Authors:** Anton Haase, Saša Bajt, Philipp Hönicke, Victor Soltwisch, Frank Scholze

**Affiliations:** a Physikalisch-Technische Bundesanstalt (PTB), Abbestrasse 2-12, 10587 Berlin, Germany; b Photon Science, DESY, Notkestrasse 85, 22607 Hamburg, Germany

**Keywords:** multilayers, water window, metrology, roughness, interdiffusion

## Abstract

Cr/Sc multilayer systems can be used as near-normal incidence mirrors for the water window spectral range. It is shown that a detailed characterization of these multilayer systems with 400 bilayers of Cr and Sc, each with individual layer thicknesses <1 nm, is attainable by the combination of several analytical techniques. EUV and X-ray reflectance measurements, resonant EUV reflectance across the Sc *L* edge, and X-ray standing wave fluorescence measurements were used. The parameters of the multilayer model were determined *via* a particle-swarm optimizer and validated using a Markov chain Monte Carlo maximum-likelihood approach. For the determination of the interface roughness, diffuse scattering measurements were conducted.

## Abstract 2

This paper reports the characterization of subnanometre Cr/Sc multilayers through the application of several analytical experiments. A combined analysis is shown and verified by Markov chain Monte Carlo sampling.

## 1. Introduction   

The wavelength range of the so-called ‘water window’ between 2.3 and 4.4 nm is of special interest, because radiation in this spectral range shows low absorption in water, while it is absorbed by many elements naturally occurring in organic molecules such as proteins (Kirz *et al.*, 1995 ▸). This allows the study of biological systems in their native environment (water), where many proteins are biologically active. In addition to the short wavelength required to achieve high-resolution imaging of such samples, one also needs sufficient intensity, which can be achieved with high-reflectance optical elements (Hertz *et al.*, 1999 ▸; Legall *et al.*, 2012 ▸).

The strong absorption of soft X-ray radiation in most materials poses a challenge in the fabrication of such optics. Refractive optical elements are not available owing to the high absorption in solids. The same holds for reflective optical elements close to normal incidence, where the reflectivities from a single surface are well below 10^−4^ for all materials (Henke *et al.*, 1993 ▸). A candidate system for building highly reflective mirrors for short wavelengths is a layered structure with alternating materials of significantly different refractive indices (Spiller, 1972 ▸). Such multiple repeated bilayer systems constitute an artificial one-dimensional Bragg crystal. Their layer layout, more specifically the total layer thickness *D* of a single layer period, is intrinsically related to the desired peak reflectance wavelength and incidence angle. Such systems are well established as mirrors for an EUV wavelength of 13.5 nm, where they reflect more than 60% of the radiation close to normal incidence with a choice of Mo and Si as layer materials (Barbee *et al.*, 1985 ▸; Stearns *et al.*, 1991 ▸). By applying additional interface shaping techniques and adding barrier layers to prevent interdiffusion, reflectivities of above 70%, close to the theoretical limit, are achievable (Bajt *et al.*, 2002 ▸).

Theoretical calculations show that constructing multilayer mirrors in the water window spectral range for normal incidence allows peak reflectivities above 50% (Schäfers *et al.*, 1998 ▸). A typical choice of materials for these bilayer systems is Cr and Sc for wavelengths above 3.1 nm (Salashchenko & Shamov, 1997 ▸; Schäfers *et al.*, 1998 ▸). The proximity to the Sc *L* edge causes the required significant difference in the refractive index due to anomalous dispersion while maintaining relatively low absorption. In order to function as a one-dimensional Bragg crystal, those layer systems demand a high quality of the layer interfaces. Chemically abrupt and smooth interfaces are required to reach high reflectivities and to minimize loss processes such as diffuse scattering or contrast reduction due to interdiffusion. This requirement becomes even more stringent when moving towards shorter wavelengths owing to the necessary reduction in layer thickness for fulfilling the Bragg condition. The relative influence of interface morphology and interdiffusion as loss mechanisms for peak reflectance rises in importance compared with established Mo/Si multilayer systems with significantly larger layer thicknesses. The measured peak reflectance of state-of-the-art Cr/Sc multilayer systems designed for the above specifications scores at reflectivities below 20%, less than half of the theoretically possible value (Eriksson *et al.*, 2003 ▸; Yulin *et al.*, 2004 ▸).

Roughness causes diffuse scattering out of the specular beam direction (Sinha, 1994 ▸). Interdiffusion, on the other hand, reduces the optical contrast, *i.e.* the local difference in the refractive index, thereby reducing the reflectance at each interface (Nakajima *et al.*, 1988 ▸). In order to gain a deeper understanding of the interface morphology, a characterization of the individual contributions of interface diffusion and roughness is required. Both lead to a damping of the peak reflectance (Croce & Névot, 1976 ▸). The inspection of diffusely scattered light is a natural tool for the investigation of the roughness at the interfaces. At-wavelength in-plane diffuse scattering contains information on the interface morphology. An important advantage of this analysis is that the angle of incidence is close to the surface normal, in contrast to established methods for interface characterization of thin films such as grazing-incidence small-angle X-ray scattering (Levine *et al.*, 1989 ▸). This allows the investigation of the multilayer stack locally even for strongly curved surfaces, *e.g.* in the case of focusing optics.

Standard characterization methods such as EUV reflectance and X-ray reflectance (XRR) with simple binary layer models have proven useful for the characterization of similar multilayer systems, *e.g.* Mo/Si mirrors designed for 13.5 nm wavelength (Lim *et al.*, 2001 ▸; Bajt *et al.*, 2001 ▸; Braun *et al.*, 2002 ▸; Barbee *et al.*, 1985 ▸). However, these systems typically have thicknesses of 3–4 nm for the individual Mo and Si layers. With the efforts to reduce the peak reflectance wavelength, those methods fail to yield consistent information in the framework of simple models that describe the measured reflectivities. This has already been observed in the case of La/B multilayer mirrors designed for peak reflectivities at 6.7 nm wavelength (Yakunin *et al.*, 2014 ▸).

The reason for this might be an increase in disturbances at the interfaces, which potentially break the symmetry condition. This needs to be taken into account explicitly in the model and leaves the simple binary approach with Névot–Croce damping factors as an insufficient description of the physical situation. However, the increased number of parameters required to describe such a realistic model also requires more data (information) from analytical measurements. We thus apply a set of different experimental methods to obtain a consistent reconstruction of the multilayer structure with a non-destructive approach. We demonstrate that, in the case of layer systems in the subnanometre region, a combined analysis of these experiments is required. We describe the layer system with graded interface profiles to account for the intermixing of the two materials. The validation of the derived model is conducted by applying a Markov chain Monte Carlo sampler.

## 2. Experimental details   

The Cr/Sc multilayer sample was prepared at the DESY X-ray multilayer laboratory by DC magnetron sputtering. The deposition was performed at 0.133 Pa ultra-high-purity Ar (99.999%) and a power of 200 W for both Sc and Cr sputtering targets. The multilayer is composed of alternating layers of Cr and Sc with periodic replication of the bilayer stack by *N* = 400 times. The substrate is a superpolished Si wafer piece. The sample dimensions measure approximately 20 × 20 mm. More details can be found elsewhere (Prasciolu *et al.*, 2014 ▸). The multilayer mirror was designed to reflect radiation in the water window energetically, just below the Sc *L* edge, close to a 3.1 nm wavelength at an angle of incidence of 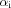 = 88.5°.

**Figure 1:**
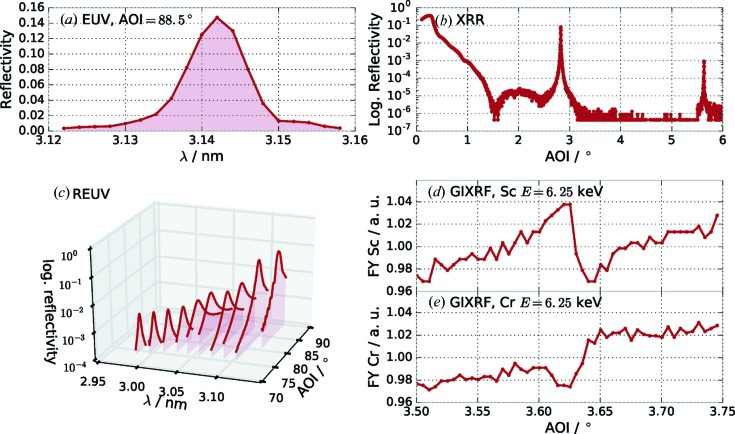
Data of the various specular and fluorescence measurements from the Cr/Sc multilayer sample. (*a*) EUV reflectance at an angle of incidence of 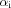 = 88.5° from the surface. (*b*) XRR measured with Cu *K*α radiation. (*c*) REUV reflectance across the Sc *L* edge. Several angular reflectance scans were performed at selected wavelengths across the Sc *L* edge. (*d*) and (*e*) XSW fluorescence recorded across the first Bragg peak by varying the angle of incidence for the Sc signal (*d*) and the Cr signal (*e*). Both curves were recorded simultaneously at an excitation energy of *E* = 6.25 keV.

The characterization *via* XRR (Fig. 1 ▸ *b*) was conducted at DESY using a laboratory-based X-ray diffractometer (X’Pert PRO MRD, Panalytical). It is equipped with a high-resolution goniometer and uses Cu *K*α radiation. The XRR intensities were recorded using a PIXcel counting detector. The dynamic range achieved in the measurements extended down to a reflectance of 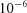 for grazing angles of incidence of 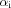 = 0° to 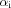 = 3°. Owing to the short period of the multilayer sample, only two Bragg peaks could be observed in this angular range. All higher-order peaks were below the detection threshold of 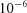 in reflected intensity.

All other experiments were conducted in the laboratory of the Physikalisch-Technische Bundesanstalt (PTB) at the electron storage ring BESSY II in Berlin-Adlershof. The EUV reflectance (Fig. 1 ▸ *a*) and resonant EUV (REUV) reflectance measurements (*cf.* Fig. 1 ▸ *c*) were performed in the ellipso-scatterometer (Soltwisch *et al.*, 2015 ▸) at the soft X-ray radiometry beamline (Beckhoff *et al.*, 2009 ▸) under ultra-high-vacuum conditions. The accessible spectral region at this beamline ranges from 0.8 to 25 nm. The samples were mounted on a six-axis goniometer sample holder. The movable detector arm in combination with the goniometer allows measurements in in-plane and out-of-plane geometry. For the REUV reflectance measurements across the Sc *L* edge the wavelength was kept fixed while performing a specular angular reflectance scan (Θ/2Θ scan). The X-ray standing wave experiments (XSW) and grazing-incidence X-ray fluorescence experiments (GIXRF) were conducted at the four-crystal monochromator beamline (Krumrey & Ulm, 2001 ▸), where energies up to 10 keV, well above the *K*-absorption edges of Sc and Cr, are accessible. The experimental setup used for the XSW experiments is a dedicated setup for reference-free X-ray fluorescence (Lubeck *et al.*, 2013 ▸). The fluorescence data were taken at an excitation photon energy of *E* = 6.25 keV, above the Cr edge. The relative fluorescence yield from the Sc and Cr *K* edges, respectively, is shown in Figs. 1 ▸(*d*) and 1 ▸(*e*). The grazing angle of incidence was varied across the resonance condition for the first Bragg peak to excite the standing wavefield inside the multilayer and to gain depth-dependent information as the XSW nodes are shifted in depth by scanning the incident angle (Hönicke *et al.*, 2010 ▸).

The diffuse scattering measurements were performed with a detector angle of 3° with respect to the incoming beam, while rocking the sample from 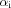 = 88.5 to 82.0° (which corresponds to normal incidence angles from 1.5 to 8.0°) in steps of 0.1° and tuning the wavelength from λ = 3.0 to 3.4 nm at each angular position with a step width of 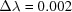
 nm. Diffuse scattering measurements close to near-normal incidence allow local measurements because of the small beam spot size on the sample resulting from a beam diameter of approximately 1 × 1 mm perpendicular to the beam. This measurement technique allows a reciprocal-space map of the diffuse scattering distribution to be obtained (the recorded data are shown below in Fig. 8 of §7).

## 3. Theoretical background   

### 3.1. Matrix formalism: EUV, REUV, XRR   

The EUV and XRR are calculated on the basis of the well established matrix formalism (Born & Wolf, 1965 ▸). The ideal reflection 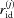 and transmission 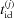 at each interface *j* (not considering roughness) are given by the Fresnel coefficients: 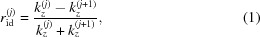


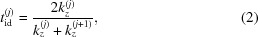
where 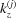 is the complex *z* component of the incident wavevector 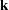 at the *j*th interface. Its value is calculated according to Snell’s law at each interface taking into account the complex indices of refraction 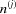. In order to account for the roughness- and interdiffusion-induced loss of specular reflectance, modified Fresnel coefficients based on a Névot–Croce factor using σ, which accounts for interdiffusion and roughness, are considered at each interface (Croce & Névot, 1976 ▸). We assume the interface mean-square roughness to be identical at each interface, *i.e.* 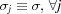. Considering the different roughnesses at each interface would increase the number of variable parameters by the quantity of interfaces and thus lead to an ill defined model. The analysis of the diffuse scattering from Mo/Si multilayer systems has shown a high correlation of the interface roughness throughout the stack (Haase *et al.*, 2014 ▸), which justifies the assumption of identical roughness made here. With this approximation the modified Fresnel coefficients are given by 
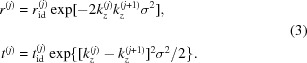
The electric fields at each interface are then related to the fields at the next interface through the field propagation matrix 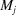 (Born & Wolf, 1965 ▸; Mikulík, 1997 ▸): 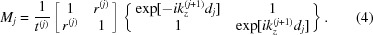
The fields inside the multilayer stack, as well as inside the substrate and the vacuum, are given by equation (5): 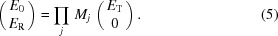
The total wavefield is represented by a two-dimensional vector. The upper component describes the amplitude of the wave propagating towards the substrate and the lower component describes the reflected wave amplitude propagating towards the vacuum. There is no radiation incident from the substrate side towards the vacuum. The iterative application of the field propagation matrix yields the electric field amplitudes *E* at each interface for both propagation directions, with the known incoming wave amplitude 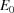.

The components 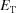 and 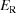 describe the transmitted and reflected field amplitudes inside the vacuum and the substrate, respectively. Normalized reflectance *R* and transmittivity *T* values for the EUV, REUV and XRR measurements are obtained from the field calculation by dividing the calculated values by the initial field amplitude 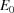: 
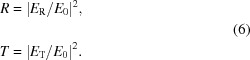



### 3.2. X-ray standing wave fluorescence analysis   

The calculation of the relative fluorescence signal 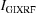 for either element in the multilayer stack is performed by discrete numerical integration of the product of the total electric field intensities, 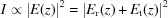, where 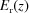 and 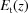 are the reflected and transmitted field amplitudes inside the layer specified by the vertical coordinate *z*, with the relative density profile of the elements, 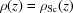 or 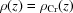, along the surface normal of the sample, *i.e.*

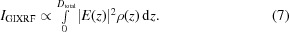
Here the total thickness 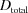 of the multilayer stack is given by 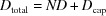, *N* being the number of multilayer periods, *D* the thickness of a single period and 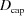 the total thickness of all capping layers, and *z* is the coordinate along the surface normal with respect to the substrate surface. This calculation is an approximation, since absorption effects of the fluorescence radiation leaving the sample are omitted. However, in the case of a relative comparison of the signal, as performed here, this approximation is justified. For the numerical integration, the total electric field 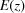 is evaluated on a sufficiently fine grid across all periods of the multilayer including the interfaces according to equation (5).

### 3.3. Distorted-wave Born approximation   

The theoretical analysis of the diffuse EUV scattering data obtained has been conducted on the basis of the distorted-wave Born approximation (DWBA) (Holý & Baumbach, 1994 ▸; Holy *et al.*, 1993 ▸). Here, the interface roughness is considered to be a small distortion of the solution of the perfect multilayer system. We apply that to diffuse scattering measurements at near-normal incidence, where dynamic effects become important and need to be considered in order to obtain the power spectral density (PSD) of the interface morphology. Our approach is described in detail by Haase *et al.* (2014 ▸). Following from equations (5) and (6), the explicit transmitted and reflected fields at the interfaces are given by 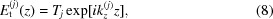


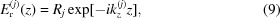
where 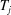 and 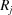 are the transmitted and reflected field amplitudes at each interface, respectively. The fields are calculated on the basis of the matrix formalism described above using the undisturbed system, *i.e.* the ideal Fresnel coefficients instead of the modified coefficients already including a correction for roughness. The solution serves as the undisturbed wavefield entering the DWBA calculation. The diffuse scattering intensity into the solid angle dΩ is then given by the differential cross section
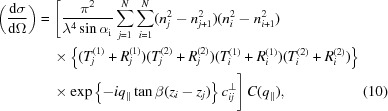
taking into account all first-order dynamic effects. Here, 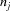 represents the complex refractive index inside each layer, 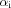 represents the angle of incidence from the surface, λ represents the wavelength of the impinging radiation, and β indicates the angle at which correlated roughness replicates from interface to interface throughout the stack and 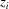 the position of the interface, both with respect to the surface normal. The leading term of equation (10) contained within square brackets can be considered as a multilayer enhancement factor separating the contributions from the periodicity of the sample and the vertical correlations from the roughness at the interfaces isolated in the two-dimensional PSD, 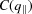 (Haase *et al.*, 2014 ▸). The scattering distribution is calculated in so-called reciprocal-space coordinates, *i.e.* the momentum transfer vector **q**, given by 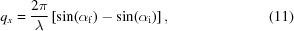


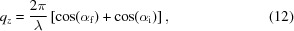
where 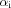 is the angle of incidence from the normal and 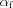 is the scattering angle. All measurements were performed in the plane spanned by the incoming beam and the surface normal, *i.e.* all measurements were taken at vanishing azimuthal angles. Consequently, 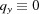 in our case, such that the component of the wavevector transfer parallel to the surface and interfaces is given by 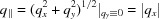. The roughness properties of the interfaces are incorporated in the replication factor 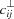 (Spiller *et al.*, 1993 ▸) and in the effective one-dimensional PSD 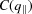. Several PSD models exist in the literature, *e.g.* by Sinha *et al.* (1988 ▸), for single rough interfaces or surfaces. Owing to the low thickness of the individual layers we make the approximation of identical statistical properties of each interface with respect to the roughness, *i.e.* we assume identical PSDs. A separate treatment of each interface would be theoretically possible. However, this would pose an ill defined model since individually different interfaces cannot be distinguished methodologically with scattering techniques. The high degree of vertical correlation was confirmed for our samples through the formation of a narrow Bragg sheet in the data presented below in Fig. 8. We follow the definition of de Boer and co-workers (de Boer *et al.*, 1994 ▸; de Boer, 1995 ▸), which yields a closed analytic form of the PSD for a fractal roughness model: 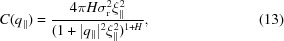
where 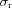 is the root-mean-square roughness, *H* is the Hurst factor describing the jaggedness of the interface and 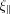 is the in-plane correlation length. The replication factor 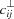 is given by 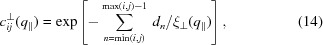
where 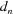 is the thickness of the *n*th layer and 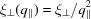 is a spatial frequency dependent vertical correlation length at which the replication factor has decreased to 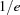.

## 4. Improved model for multiparameter reconstruction   

**Figure 2:**
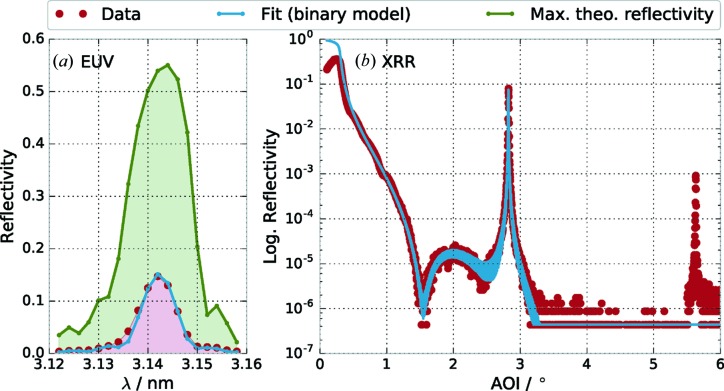
(*a*) Fitted experimental EUV reflectance curves across the wavelength of the radiation impinging at 1.5° from normal, based on the binary model. The green curve shows the maximum possible reflectance in the water window assuming a perfect multilayer system without roughness or interdiffusion. (*b*) The optimal model based on the analysis of the EUV reflectance (*cf.* Fig. 2 ▸ *a*) shows a clear mismatch with the measured XRR curve in the second Bragg peak.

To better illustrate the requirement of an improved model, we have conducted an analysis based on the standard binary model of a Cr/Sc multilayer. The analysis was based on the EUV data shown in Fig. 1 ▸(*a*). The results are shown in Fig. 2 ▸ in comparison with the expected XRR curve from this optimization result and the theoretically achievable maximum reflectance without any roughness or interdiffusion. The Sc to Cr ratio was found to be 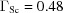 with roughness and interdiffusion considered *via* a Névot–Croce factor using σ = 0.385 nm. The individual layer thicknesses for this sample were fitted to be 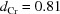
 nm and 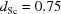
 nm. While the EUV reflectance curve (*cf.* Fig. 2 ▸ *a*) shows excellent agreement with the measured data, there is a significant offset in the case of the XRR measurement with the (binary) model derived from the EUV reflectance experiment. Even a combined analysis could not yield a consistent result, since the Névot–Croce factor required to reduce the theoretical EUV reflectance down to the measured level could not be brought into agreement with the XRR curve (result not shown here). In a strictly binary model with a layer thickness ratio of 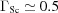, the second Bragg peak is additionally suppressed for symmetry reasons. Thus, there is a clear mismatch with the experimental observation as seen in the comparison of the fitted and measured XRR curves in Fig. 2 ▸.

**Figure 3:**
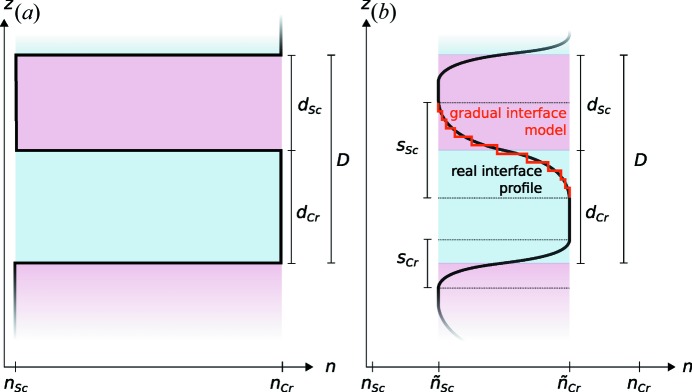
(*a*) Binary Cr/Sc multilayer model with total period thickness *D* and the individual layer thicknesses 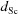 and 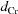. (*b*) Model with explicit gradual interfaces following a sinusoidal profile. The ideal interface profile is approximated through discrete sublayers as indicated in red, forming the actual gradual interface profile entering the electric field calculations. The thickness of the interdiffusion zones can differ for the top and bottom interface in each period. Their total thicknesses are given by 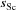 and 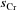. The effective index of refraction for each layer is given by 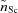 and 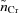, respectively.

Instead of the simple binary layer model, a gradual interface model is introduced to better reflect the electron-density profile due to interdiffusion. A corresponding profile is shown in Fig. 3 ▸ for illustration in comparison with the binary model.

The calculation of the electromagnetic fields is then conducted on the basis of this model with the matrix formalism introduced in the preceding section. The interface region with sinusoidal profiles is sampled with a fixed number of equally spaced points in the **z** direction, effectively creating a region of thin sublayers with a gradually changing index of refraction. The parameters of our multilayer model are listed in Table 1 ▸, where *D* is the full period thickness, 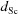 and 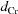 are the nominal layer thicknesses of the Cr and Sc layers as indicated in Fig. 3 ▸, and 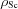 and 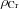 are their respective densities with respect to their bulk densities, 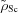 = 2.989 g cm^−3^ and 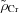 = 7.19 g cm^−3^ (Henke *et al.*, 1993 ▸). The parameters 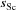 and 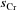 describe the full width of the interdiffusion layers as shown in Fig. 3 ▸. To take into account intermixing extending across the full period, we introduced an intermixing parameter η. The effective indices of refraction of the individual Cr and Sc layers are then given through 
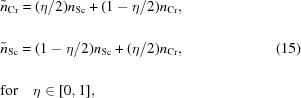
where 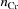 and 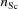 are the tabulated values (Henke *et al.*, 1993 ▸) with densities 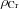 and 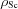. The loss of specular reflectance due to roughness-induced scattering is considered through the Névot–Croce factor using 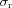 identical at each interface for the reasons described in the previous section. To improve the optimization procedure and to reduce correlations between individual parameters, we have selected some effective parameters as defined in Table 1 ▸. The parameter 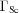 indicates the portion of the Sc layer thickness with respect to the full period thickness *D*; 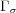 describes the asymmetry of the widths of the sinusoidal profiles at the Cr/Sc and Sc/Cr interfaces and is limited to the interval 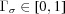. Note that 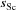 and 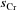 are half periods of the sinus functions used to describe the interface profiles. Therefore the condition 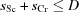 holds.

## 5. Combined analysis of EUV, REUV, XRR and GIXRF   

The reconstruction of the multilayer structure was conducted with a series of experiments. The minimization functional of the combined analysis of the optimization problem 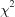 is defined as the total sum of the reduced least-squares functionals for each experiment, 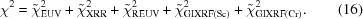
Here each of the reduced functionals is defined as 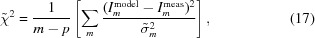
with *m* being the number of measurement points in each experiment and *p* the number of parameters for the model. Statistical and systematic uncertainties for each data point are included in 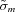. The definition of equation (16) ensures that all experiments are weighted equally considering their respective uncertainties.

The minimization is performed with a particle-swarm optimizer (PSO) (Kennedy, 2010 ▸). In the case of an optimization problem with many local minima, this provides an advantage with respect to gradient methods, where the fit result is dependent on the choice of starting values. Similar approaches employing genetic algorithms for solving the inverse problem of scatterometry can be found in the literature (Del Río *et al.*, 2000 ▸). In the case of the model parametrization given in §4, the choice of the parameter intervals is defined either by physical plausibility or by the fact that the parameter is intrinsically defined in a certain interval, the same as for the intermixing η, for example. The intervals used in our analysis are listed in Table 1 ▸.

The PSO analysis was conducted for each experiment individually and for the combination of all experiments. In the case of the XRR measurements, only the first and second Bragg peaks were considered for the combined analysis. The region in-between mainly reflects the top surface layers, *i.e.* capping layers, and potential surface contamination layers, which were analysed separately exclusively on the basis of the XRR data. The results were added as fixed surface layers to the model for all theoretical calculations of all experiments. The analysis of the GIXRF experiment was based on the fluorescence data at an excitation photon energy of 6.25 keV for the Sc *K* and Cr *K* lines by spectral deconvolution using detector response functions.

## 6. Validation with Markov chain Monte Carlo sampling and discussion   

The solution of the inverse problem based on the particle-swarm optimization technique ideally delivers the global minimum of the total 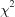 functional in the specified parameter space. However, no information is obtained about the sensitivity of an experiment with respect to certain aspects of the model, *i.e.* specific parameters. As an example, one might consider the case of an EUV reflectance experiment, where the influence of the interdiffusion layer asymmetry on the expected reflectance curve is negligible. The model reflects this geometry through the parameter 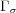. Most likely, an optimal choice for 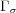 minimizing 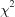 exists and can be found by using the PSO. Nevertheless, varying the parameter 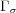 causes only marginally larger 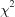, resulting in a limited credibility and validity of this parameter and leaving it essentially undefined on the basis of the available data. To solve this issue and quantify confidence intervals for each parameter in each experiment, we apply a Markov chain Monte Carlo (MCMC) sampling technique. The likelihood of the model describing the actual sample based on the data available is given by 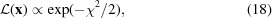
where 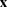 is the set of parameters of the model and 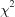 is the total 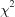 for the validation of the combined analysis [*cf.* equation (16)] or the reduced 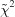 for the validation of the individual experiments according to the definition in equation (17). We employ an existing Python-based implementation of this sampling technique (Foreman-Mackey *et al.*, 2013 ▸) to numerically sample the likelihood functional in equation (18). As a starting point we use the optimum parameter set obtained as a result of the particle-swarm optimization.

Consequently, in addition to fitting the data with a PSO, we verified each result using the MCMC method described above to evaluate the confidence intervals for each parameter. The two-step process, *i.e.* the PSO fitting procedure followed by the MCMC sampling, was conducted for each standalone experiment as well as for the combined optimization problem stated in equation (16). The results are compiled in Table 2 ▸. The confidence intervals were calculated by evaluating the probability distribution as a result of the MCMC procedure for each parameter around its PSO fit results. The confidence intervals given here represent percentiles of the number of samples found in the interval defined by the upper and lower bounds used for the PSO procedure for each parameter. In the case of a centred Gaussian distribution, percentiles of 2.3 and 97.8% of the integrated number of samples forming the distribution mark the interval of four times the standard deviation, *i.e.* 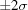 in statistical terms. Owing to potential asymmetries in the actual distributions found by the MCMC method, explicit upper and lower bounds of the confidence intervals are given in Table 2 ▸, based on these percentiles. The best model value is based on the PSO fit result and is refined by the MCMC sampling by calculating the mean value, *i.e.* the 50% percentile, of the distribution of samples following the MCMC procedure. The best model is thus the result of a two-step optimization routine starting with a PSO analysis and sampling based on the resulting values to evaluate the distribution according to equation (18).

**Figure 4:**
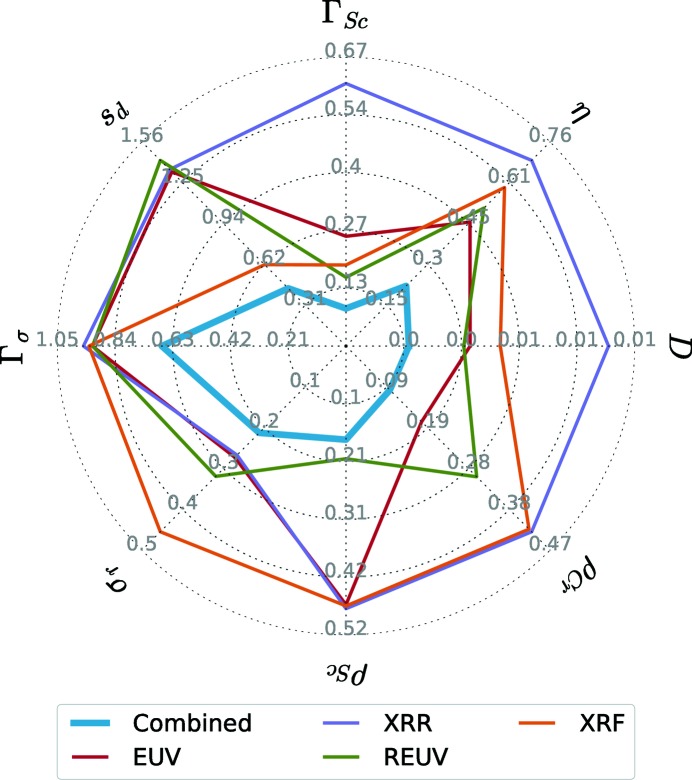
Visual representation of the total confidence intervals for each of the parameters with respect to each of the individual experiments as well as the combined analysis.

The confidence intervals of each experimental method differ significantly depending on the parameter. To better demonstrate the different sensitivities for the model parameters depending on the experimental method, we have illustrated each confidence interval in Fig. 4 ▸.

It is worth noting that the confidence interval for the combined analysis is significantly smaller than those for the individual experiments. This is especially true for the parameter 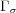 describing the asymmetry of the interdiffusion layers. Within each of the individual experiments this parameter has a large uncertainty, whereas the combined analysis delivers a significant result of a clearly asymmetric interdiffusion layer thickness.

Another conclusion drawn from the confidence intervals of each of the individual experiments shown in Fig. 4 ▸ is that each method scores best for at least one of the model parameters. Omitting one experiment from the combined analysis would therefore lead to worse confidence intervals. However, determining whether a unique solution could still be found would require an MCMC analysis of all possible combinations of all experiments.

**Figure 5:**
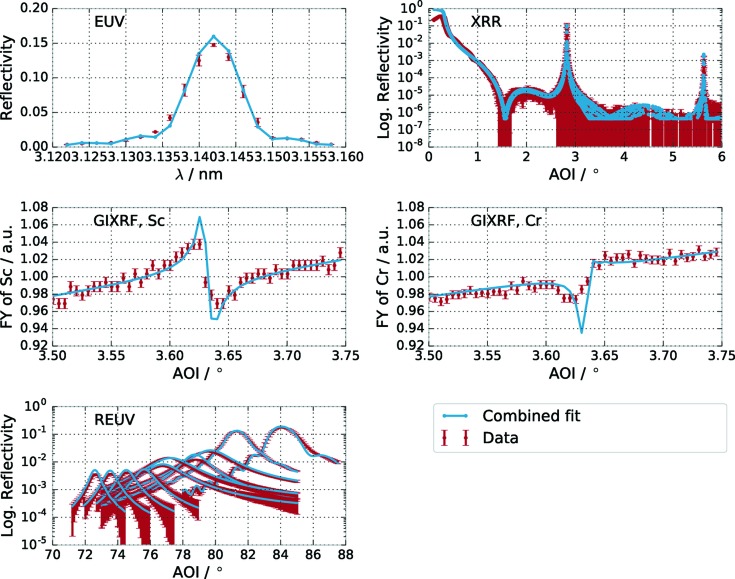
Measured and calculated reflectance and intensity curves for the optimized parameters with the combined analysis of all experiments as listed in Table 1 ▸.

The best-fit result based on the two-step optimization procedure of the combined data set of all experiments is shown in Fig. 5 ▸ together with the experimental data. The theoretical calculations based on the above model and the experimental data show good agreement. Nevertheless, differences can be observed. The reason lies in the fact that the model is potentially still too ideal. Small variations during the deposition process, for example, could lead to imperfections, which are not described in a strictly periodic model. However, including these by explicitly breaking the periodicity would again lead to an ill defined model with a vastly increased number of parameters and is thus not practical. Another reason is the deviation in the homogeneity of the sample, *e.g.* a varying period across the sample, which causes mismatches if the measurement position varies slightly between the different experimental setups. The latter effects were considered in the uncertainties of the individual measurements by measuring the EUV reflectivity at positions 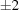
 mm from the centre position and fitting the model. The result was a 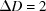
 pm shift in the period over 4 mm across the sample.

**Figure 6:**
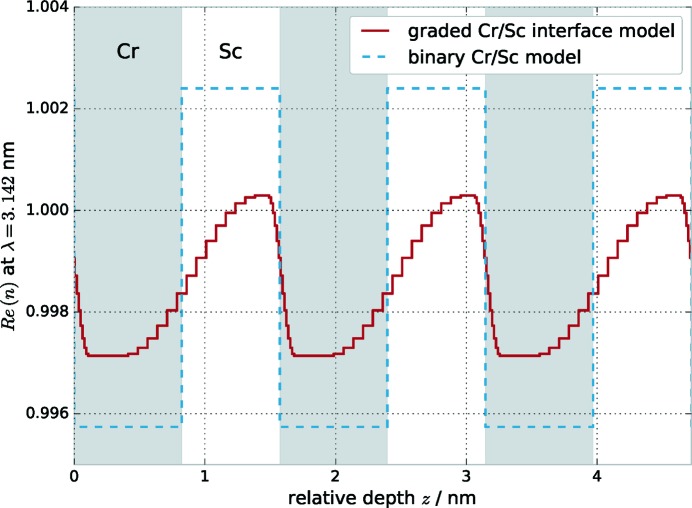
Real part of the index of refraction *n* based on the results of the optimized parameters listed in Table 2 ▸ for the combined analysis for a selected wavelength. The gradual interface model is shown in direct comparison with the binary model optimized for the EUV reflectance curve over three full periods. The resulting strong asymmetry in the width of the interface regions is clearly visible (see text). The grey and white shaded areas indicate the Cr and Sc layers, respectively, for the binary model.

The resulting depth dependence of the index of refraction is shown in Fig. 6 ▸ together with the initial binary model for comparison.

The most remarkable result of the combined analysis is the strong asymmetry of the interdiffusion layers. This can only be shown by the combination of all analytical experiments conducted here, as can be seen from the confidence intervals in Fig. 4 ▸ as well as the values in Table 2 ▸. A possible explanation for this asymmetry is the deposition process through magnetron sputtering. The elements Cr and Sc have different mass and thus different momentum when deposited onto each other. A similar effect is known from the deposition of Mo/Si multilayer systems, where the heavier Mo shows higher penetration into the Si layer than *vice versa* (Petford-Long *et al.*, 1987 ▸). In the case of Cr/Sc multilayers, the Cr is heavier and thus has higher momentum, leading to a broader interdiffusion layer. The validation using the MCMC procedure also yields possible correlations between single parameters of the model.

## 7. Diffuse scatter   

**Figure 7:**
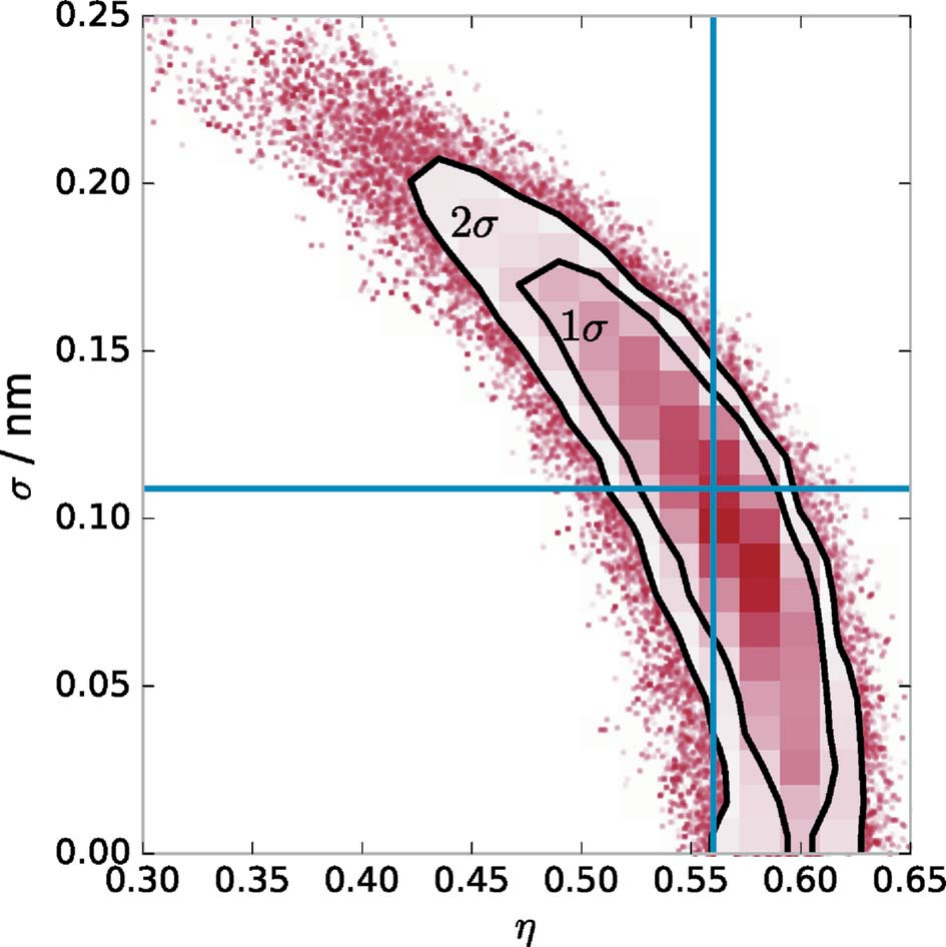
Correlation of the projected 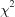 surface onto the parameter pair 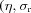) by visualization of the position of the MCMC samples in the reduced parameter space. The strong correlation of the two parameters in the optimal solution, which is indicated by blue solid lines, is clearly visible. The percentiles corresponding to one and two standard deviations σ are indicated by the black contour lines.

**Figure 8:**
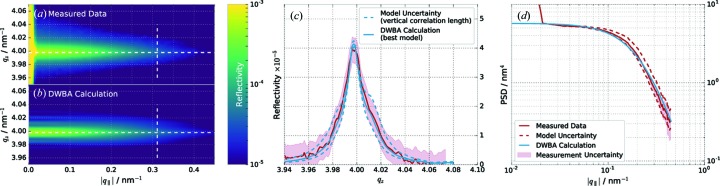
(*a*) Diffuse scattering measurement in *q*-space representation and log scale. (*b*) DWBA calculation of the optimal PSD model based on the electron-density profile with the multilayer parameters for the combined analysis listed in Table 2 ▸. (*c*) Vertical cut at the indicated white dashed cut positions in (*a*) and (*b*). The blue dashed lines show two limiting cases for the value of the vertical correlation length. The result is the model uncertainty in the PSD. (*d*) Comparison of the extracted effective PSDs from the diffuse scattering measurement (Measured Data) shown in (*a*) and the DWBA calculation of (*b*) at the horizontal cut positions indicated by the white dashed lines. The uncertainty interval for the extracted PSD is shown by the two dashed PSD profiles (see main text).

Even in the case of the data, methods and model presented here, the combined analysis leaves a correlation between the intermixing parameter η and the roughness 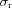, which could not be resolved (see Fig. 7 ▸). This means that none of the methods, not even the combined analysis, contains sufficient information to deduce an unambiguous result for the roughness or intermixing. Intermixing alone merely reduces optical contrast, whereas interface roughness causes diffuse scattering. One should be able to distinguish between the two through the measurement of the diffuse scattering. The distribution of the off-specular scattering with respect to the scattering angle and wavelength provides additional information on the vertical and lateral correlation of spatial roughness frequencies. The latter is described by the PSD. We conducted a diffuse scattering experiment as described in §2. The analysis was based on the DWBA formalism outlined in §3. Parts (*a*) and (*b*) of Fig. 8 ▸ show the measured reciprocal-space map in direct comparison with the best model found within the DWBA approach.

The formation of a narrow Bragg sheet (Haase *et al.*, 2014 ▸; Salditt *et al.*, 1994 ▸) confirms the high degree of roughness correlation and thereby justifies the approximations made in §3 for identical roughness properties at each interface. To deduce the effective PSD shown in Fig. 8 ▸(*c*), we have taken a cut along the Bragg sheet as indicated by the horizontal white dashed lines in the reciprocal-space maps. We divided the extracted scattering intensity by the multilayer enhancement factor in equation (10), leaving the contribution of the effective PSD 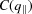 to the diffuse scattering. This requires that the vertical correlation factor 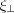 be determined first, which enters the calculation of the multilayer enhancement factor through the replication factor in equation (14). Owing to the very high computational cost of an MCMC procedure, we have instead calculated two limiting cases of the vertical correlation. This was done by analysing the width of the Bragg sheet at the vertical white dashed cut positions, indicated in Figs. 8 ▸(*a*) and 8 ▸(*b*), and comparing the simulated intensity distribution with the measurement uncertainty. The two limiting cases are shown in Fig. 8 ▸(*d*) (red dashed curves), including the best model (solid blue curve) in comparison with the measured data (solid red curve). Proceeding from here, we have evaluated the measured PSD with the corresponding multilayer enhancement factor as described above. The two limiting cases are shown as blue dashed curves in Fig. 8 ▸(*c*), including the PSD deduced from the best model value for 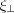 as a solid red curve. The root-mean-square (r.m.s.) roughness deduced from these PSDs is given by the two-dimensional integral as 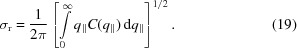
The uncertainty of the PSD due to the vertical correlation leads to an uncertainty in the r.m.s. roughness when evaluating the integral. Owing to the limited 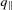 range where measurements can be taken, we have fitted the PSD model of equation (13) to the three resulting PSDs and performed the integration over the full 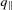 range. The deviation of the integration for the PSD model fit and the data in the available range was negligible. The best model results for the vertical replication factor and the PSD are given in Table 3 ▸, together with their uncertainties resulting from the described procedure. The best fit of the PSD model is shown in Fig. 8 ▸(*c*) as a solid blue curve.

The r.m.s. roughness value found with the analysis of the diffuse scattering is identical within its uncertainty interval to the value obtained from the combined analysis and thus confirms the intermixing and roughness parameters listed in Table 2 ▸.

## 8. Conclusions   

In conclusion, we have demonstrated a robust method to characterize ultra-thin multilayer systems with subnanometre layer thicknesses unambiguously. Layer thicknesses in the subnanometre region are necessary for near-normal incidence reflective mirrors in the water window spectral range. However, they come with the cost of increasing susceptibility to disturbances in the interfaces at the layer boundaries. This limits the achievable reflectance to values well below the theoretical threshold, creating a demand for ideally non-destructive characterization methods. The main mechanisms for diminished reflectance are interdiffusion and roughness. With these effects being of the order of the layer thickness, models based on binary layer stacks become inadequate to describe the physical situation. In order to find a proper representation of the multilayer sample, more sophisticated models with an explicit description of the gradual interdiffusion layers become necessary. This inevitably increases the number of parameters to be determined in analytical experiments. Finding an unambiguous solution is challenging and can only be achieved with a combined analysis of several non-destructive techniques.

We performed a rigorous analysis of several experimental methods to determine the model parameters representing one Cr/Sc sample. The optimal set of parameters was determined by applying a particle-swarm optimizer in conjunction with a Markov chain Monte Carlo method to verify the uniqueness of the solution and derive confidence intervals for all parameters in all experiments. The set of analytical methods we employed were EUV and X-ray reflectance, resonant EUV reflectance across the Sc *L* edge, and X-ray standing wave fluorescence at the Sc *K* and Cr *K* lines across the first Bragg peak. The analysis of each method shows different sensitivities for specific parameters of the model. The EUV reflectance shows sensitivity for the optical contrast, *i.e.* the intermixing η and the roughness 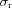. With the resonant EUV reflectance this is further improved and additional sensitivity is added with respect to the ratio of Sc and Cr as well as the total period thickness *D*. The XRR measurement, on the other hand, yields better confidence intervals for the roughness 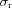 owing to the appearance of the second Bragg peak. Finally, GIXRF delivers a method to resolve the multilayer structure spatially and thus the interdiffusion layer thickness 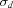 and the Sc to Cr ratio.

Within the verified confidence intervals the MCMC methods reveal a remaining correlation between the intermixing parameter and the roughness factor, which could not be resolved with the experiments in specular geometry. We therefore performed a measurement of the off-specular diffuse scattering to distinguish between the roughness and the interdiffusion. The results of these analyses reveal a high degree of roughness correlation throughout the multilayer with interface roughness values comparable to the best fit obtained in the combined analysis. With the combination of all these methods, a robust result could be derived with improved confidence intervals. Most notably, only the combined analysis can detect the asymmetry of the interdiffusion layers 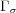. It should also be noted here that the interdiffusion width 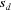 is much larger than the roughness values 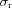. Also none of the layers were found to have the index of refraction of pure Cr or Sc. This is reflected through the non-vanishing intermixing parameter 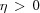. Thus, it can be concluded that, while roughness still exists, intermixing and interdiffusion are the main cause of diminished reflectance for the Cr/Sc multilayer system studied here.

## Figures and Tables

**Table 1:** Multilayer parametrization and parameter limits

Parameter	Definition	Lower bound	Upper bound
*D* (nm)	= 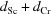	1.5	1.6
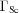	= 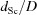	0.0	1.0
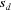 (nm)	= 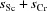	0.0	1.6
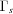	= 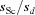	0.0	1.0
η	Layer intermixing	0.0	1.0
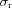 (nm)	R.m.s. roughness	0.0	0.5
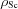	Sc density with respect to bulk density	0.5	1.0
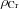	Cr density with respect to bulk density	0.5	1.0

**Table 2:** Optimized model parameters with confidence intervals derived from MCMC validation for each individual experiment and the combined analysis

Parameter	Combined	EUV	XRR	REUV	GIXRF
*D* (nm)	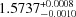	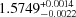	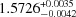	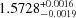	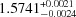
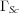	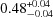	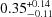	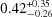	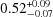	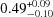
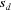 (nm)	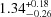	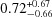	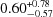	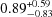	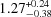
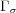	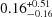	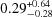	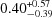	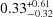	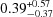
η	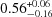	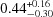	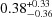	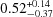	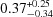
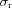 (nm)	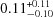	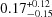	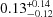	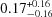	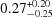
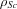	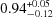	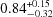	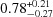	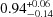	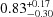
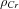	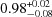	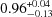	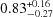	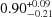	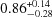

**Table 3:** Best model parameters of the PSD as a result of the diffuse scattering analysis

Parameter	Best model values
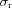 (nm)	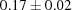
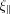 (nm)	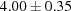
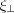 (nm^−1^)	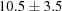
*H*	1.0
β	0.0
